# Are You Safe or Should I Go? How Perceived Trustworthiness and Probability of a Sexual Transmittable Infection Impact Activation of the Salience Network

**DOI:** 10.1523/ENEURO.0258-24.2024

**Published:** 2025-02-18

**Authors:** Alexander Wolber, Stephanie N. L. Schmidt, Brigitte Rockstroh, Daniela Mier

**Affiliations:** Department of Psychology, University of Konstanz, Konstanz 78457, Germany

**Keywords:** fMRI, HIV, risk, salience network, trustworthiness

## Abstract

Functional imaging studies indicate that both the assessment of a person as untrustworthy and the assumption that a person has a sexually transmitted infection are associated with activation in regions of the salience network. However, studies are missing that combine these aspects and investigate the perceived trustworthiness of individuals previously assessed with high or low probability of a sexually transmitted infection. During fMRI measurements, 25 participants viewed photographs of people preclassified as having high or low HIV probability and judged their trustworthiness. In a postrating, stimuli were rated for trustworthiness, attractiveness, and HIV probability. Persons preclassified as HIV− in contrast to those preclassified as HIV+ were rated more trustworthy and with lower HIV probability. Activation in medial orbitofrontal cortex was higher for those rated and preclassified as HIV− than HIV+. Based on the individual ratings, but not the preclassification, there was significantly higher activation in the insula, amygdala, anterior cingulate cortex, and nucleus accumbens in response to untrustworthy than to trustworthy faces. Activation of the salience network occurred when a person was judged as untrustworthy, but not according to a preclassification. Activation in the medial orbitofrontal cortex, a structure associated with reward, was enhanced when a person was perceived as trustworthy and also when a person was preclassified with low HIV probability. Our findings suggest that trustworthiness and HIV− perception have consistency across samples, while the perception of risk and associated activation of the salience network has restricted cross-sample consistency.

## Significance Statement

Whether a person is trustworthy or might pose a risk to one's own health must be decided in a few moments and based on limited characteristics. The salience network as an “alarm system” should be involved in these evaluative processes. This paper reports the results of neural activation in trustworthiness judgments of naturalistic stimuli of persons precategorized as HIV+ or HIV−. We find activation in medial orbitofrontal cortex for people evaluated as trustworthy and for people precategorized as HIV−. For people judged as untrustworthy, activation in the insula, amygdala, anterior cingulate cortex, and nucleus accumbens is revealed. These findings suggest a safety signal in the medial orbitofrontal cortex and an involvement of the salience network in risk detection.

## Introduction

Individuals’ motivation to engage with another person is influenced by implicit assumptions about their interaction-associated risks, based on more general assumptions about the counterpart's traits, such as their overall trustworthiness (Van’t Wout and Sanfey, 2008). According to Adolphs and Tusche (2017), such automatic perception of a counterpart as trustworthy results from the automatic comparison of visual, primarily facial cues with one's own internalized sense of a facial expression's trustworthiness and the similarity with one's own face.

The impact of trustworthiness on risk perception and behavior has become a target of research in the context of sexually transmitted infections (STI) such as HIV infection, as the implicit perception of another person as trustworthy (or not) affects the engagement in unprotected sexual interaction (Renner et al., 2012). In light of the high prevalence of risky sexual interactions despite awareness and knowledge of the risks, identifying factors (such as perceived trustworthiness) that modify risk behavior should inform preventive and instructive strategies. With this aim, the present study addressed the relationship between trustworthiness and risk appraisal, more precisely, how ratings of portrayed persons as trustworthy (or not) relate to their evaluation as being HIV carriers. Building on a negative correlation between these two ratings reported by Renner et al. (2012), Häcker et al. (2015), and Schmälzle et al. (2011), the present study used facial stimuli categorized by participants as either HIV carrier (HIV+) or not (HIV−). Whether or to what extent trustworthiness appraisal is linked to shared appraisal of faces reflecting HIV risk or rather related to individual facial appraisal of HIV risk was examined by presenting the series of facial stimuli twice. First, during the fMRI scan with the task of a yes/no decision on the trustworthiness, and secondly, after the fMRI scan, with the instruction to rate the likelihood of the portrayed individual to be an HIV carrier, their trustworthiness and attractiveness.

Hemodynamic neuroimaging (fMRI) served to substantiate the neural correlates of trustworthiness evaluation, with an emphasis on cortical and subcortical structures and circuits mediating risk processing. The salience network (SN) is a large-scale brain network responding to emotional and social cues, including threatening events, such as potential health risks (Ince et al., 2023). The SN as functional integration of dorsal anterior cingulate cortex (dACC) and fronto-insular cortex (FIC) with its connections to medial prefrontal cortex (mPFC), amygdala and substantia nigra, and ventral tegmental area (Bressler and Menon, 2010; Menon, 2011) should be involved in trustworthiness and risk evaluation. Indeed, Häcker et al. (2015) reported that the appraisal of persons as being HIV+ modified activity in, and connectivity between, SN structures, in particular of dorsal insula and mPFC. Following up on this evidence, as well as further studies revealing a prominent role of the amygdala for trustworthiness perception (Sladky et al., 2021), the present study focused on SN involvement and connectivity in trustworthiness appraisal of facial pictures selected to reflect HIV+/−. Moreover, we focused on activation of the posterior superior temporal sulcus (pSTS), a central region in facial expression processing and social cognition (Bruce and Young, 1986; Haxby and Gobbini, 2011; Said et al., 2011) which is also involved in evaluations in trust (Bzdok et al., 2011; Winston et al., 2013).

In addition to trustworthiness, the evaluation of the portrayed individuals as attractive was assessed in a postmeasurement, because attractiveness has been shown to influence perceived trust (Wilson and Eckel, 2006), and both, attractiveness and trust, are related to activation of the nucleus accumbens (Nacc) and the medial orbitofrontal cortex (mOFC; Cloutier et al., 2008; Sladky et al., 2021).

Since individual trust and HIV risk perceptions seem to be related (Barth et al., 2015), we aimed on investigating the association on the basis of a previous study that allowed precategorizing persons as (perceived) HIV− or HIV+ (Häcker et al., 2015). To this end, we used a facial risk perception task (FaRiP) to examine whether we can replicate previous findings (Renner et al., 2012; Schmälzle et al., 2019a) of an association of perceived HIV probability and trustworthiness in behavioral ratings, as well as in activation and connectivity of the salience network, according to a precategorization of the stimulus materials, and based on individual ratings. Renner et al. (2012) demonstrated high inter-rater agreement and high test–retest reliability of HIV probability ratings of the stimulus materials, suggesting the replicability.

We analyzed whether the trustworthiness ratings in the MRI session are associated with the postratings of HIV probability, as well as with attractiveness and trustworthiness, and how these postratings relate to each other. For the fMRI measurements, we expected that HIV+ compared with HIV− stimuli are related to higher activation in amygdala, insula, and mPFC and higher connectivity between insula and mPFC. The same pattern was expected for the categorization according to the individual trustworthiness ratings. Further, we examined how activation in the pSTS and ACC relates to activity during trustworthiness perception. With respect to an association between postratings and activation during the task, we assumed HIV probability ratings to correlate positively with activation in amygdala, insula, and mPFC, trustworthiness ratings to correlate negatively with activation in amygdala, insula and mPFC, and attractiveness ratings to correlate positively with activation in Nacc and mOFC.

## Materials and Methods

### Study design

The present study was part of a larger project on neural correlates of risk perception and risk behavior, involving two fMRI tasks (the here presented FaRIP task and a Balloon-Analogue-Risk Task, BART), two clinical samples (patients diagnosed with schizophrenia and patients diagnosed with alcohol use disorder), and a healthy control group in a repeated-measurement intervention/replication design. The present report focused on the results of trustworthiness appraisal by an additional sample of healthy individuals as template of average, unaffected behavioral, and fMRI responses. The study was preregistered under https://osf.io/pkbt6/ and approved by the local ethics board of the University of Konstanz.

### Participants

Thirty-four volunteers were recruited via a university platform (SONA), flyer, and word-of-mouth recommendation. Participants meeting the criteria right handedness, eligibility for MRI measurement, and no history of neurological or mental disorders (screened with a telephone version of the SCID-5-interview; Beesdo-Baum et al., 2019) were included. All participants reported high school or university degree. From initially *N* = 34 participants, one participant had to be excluded due to anatomical findings and two cancelled the second appointment. Interestingly, additional *n* = 6 had to be excluded due to an acquiescence bias; i.e., they rated all persons as trustworthy in at least one of the two MRI sessions. Thus, the final sample that was included for all analyses consisted of 25 participants (7 male, age *M* = 23.16; SD = 2.84 years). Participants received verbal and written information about study aims and design and signed written informed consent before participating. Participants received a compensation of €20 or course credits for each assessment, as well as their gain in the BART that was completed after the FaRiP task.

### Material and procedure

Participants accomplished two identical appointments scheduled 4–5 weeks apart from each other. At each appointment, participants signed written consent, received instruction, practiced the FaRiP task's trustworthiness ratings in five practice trials, and completed 2–3 practice blocks of the BART. The MRI measurements started with a structural scan and were followed by the FaRiP task and the BART. After the MRI sessions, participants completed postscan ratings of trustworthiness, attractiveness, and HIV probability. The present report refers to the FaRiP task and according postratings. The BART results are presented in Schmidt et al. (2024), and the findings on tasks reliability will be presented elsewhere.

The stimulus set had 100 pictures, with 50 pictures of women and 50 pictures of men. The pictures were selected from a previous study, in which they were rated for perceived HIV+ risk (in the original study, male/female participants evaluated only individuals of opposite gender; Häcker et al., 2015) and were taken from an online database (www.flickr.com). For both, men and women, 25 pictures were used that were classified in the previous study with HIV+ probability and 25 with HIV− probability. Importantly, this categorization relies completely on the previous study, and no information about the real health status of the stimulus persons is available. All pictures were color pictures of Caucasian individuals between 18 and 35 years of age; portraits included face and partly upper body, including clothing, socioeconomic status cues, or situational context (Häcker et al., 2015). In the present study, hetero- and bisexual participants were shown the pictures of individuals of the opposite gender, and homosexual participants were shown the pictures of individuals of their own gender. After the second appointment, we made explicit to the participants that we are not aware of an association of the appearance of a person and an actual HIV infection.

**Figure 1.**
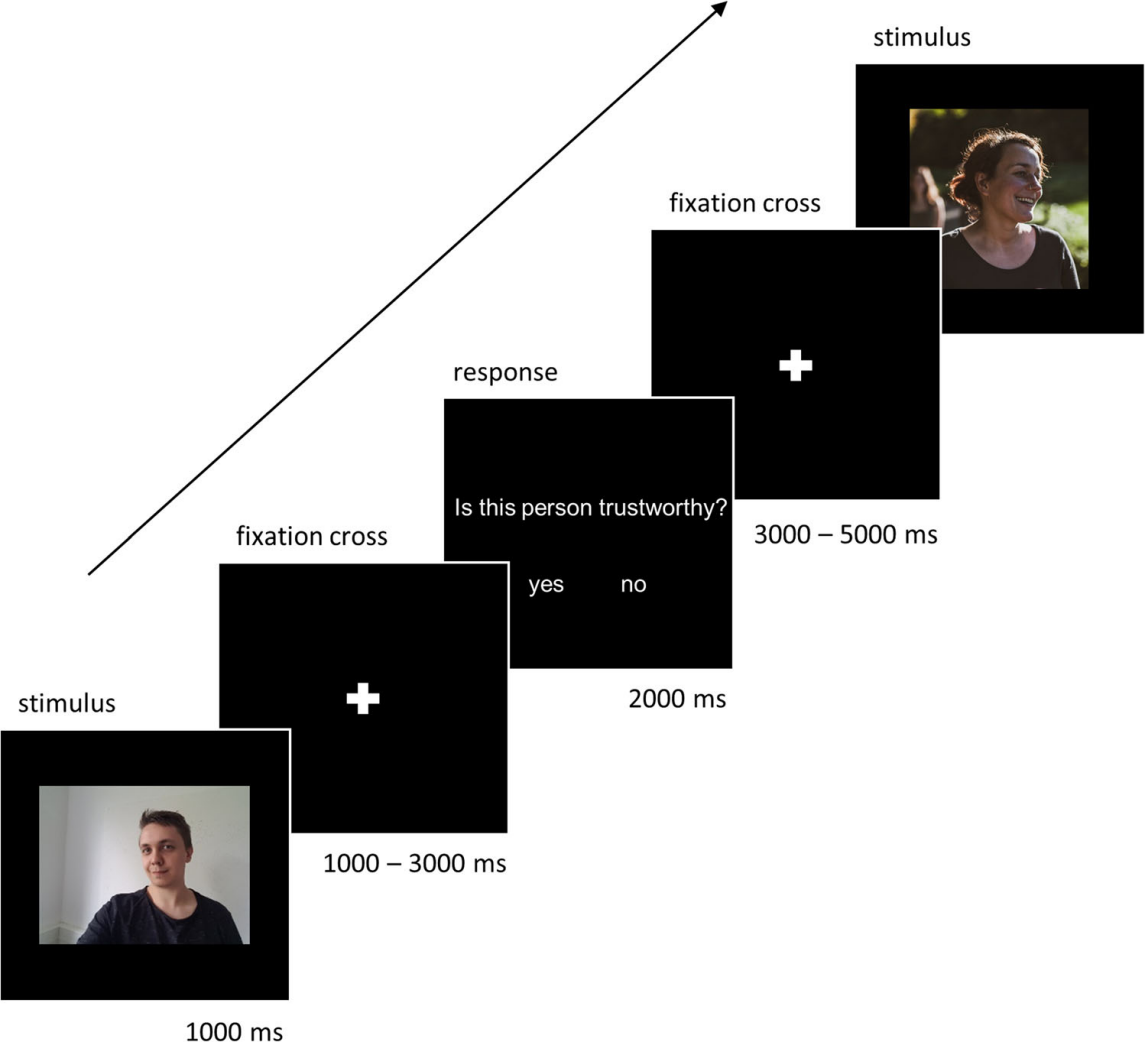
Schematic trial with exemplary pictures of the authors A.W. and D.M. that were not used in the study.

In an event-related design, across the series of 50 stimuli, each face was presented for 1,000 ms, followed by a jittered interstimulus interval with a fixation cross on a blank screen for 1,000–3,000 ms. Thereafter, the question “is this person trustworthy” appeared on the screen for 2,000 ms together with the response option “yes/no.” Participants were asked to rate each face with respect to high or low trustworthiness by pressing the left button of the response box for “yes,” and the right button for “no,” on a diamond-shaped four-button response pad (Lumina 3G Response System for fMRI – Diamond; www.cedrus.com). The FaRiP was programmed and presented using presentation (Version 21.1 Build 09.05.19, Neurobehavioral Systems; www.neurobs.com). After a 3,000–5,000 ms intertrial interval, the next face appeared (see Fig. 1 for schematic trial).

After the FaRiP task, which lasted ∼10 min, participants completed the BART and returned to a separate room for the postscan ratings of the identical series of facial stimuli, now presented on a laptop. They were instructed to judge trustworthiness, the risk of the portrayed person to be HIV carrier, and attractiveness on a 7-point Likert scale at self-paced speed.

### MRI data acquisition

We acquired fMRI data using a 3 T Siemens Magnetom Skyra (Siemens Healthcare) with syngo MR D13 software. An EPI-sequence with the following parameters was applied: TR/TE, 2,500/30 ms; flip angle, 80°; 38 interleaved axial slices (slice thickness, 3.4 mm, no gap); FOV, 218 mm; number of acquisitions, 190; matrix, 64 × 64; volume size, 3.4 × 3.4 × 3.4 mm. In addition, a high-resolution T1-weighted scan was performed with the following parameters: MPRAGE-Sequence; TR/TE, 2,500/4.06 ms; flip angle, 7°; 192 axial slices (slice thickness, 1 mm, no gap); number of volumes, 1; FOV, 256; matrix, 1 × 1 × 1.

### Data analyses

#### Behavioral indices/ratings

For the FaRiP task, we determined the number of in-scanner facial stimuli categorized as trustworthy (yes/no) and calculated the sum of both sessions. A chi^2^ test was performed for yes/no categorization relative to the HIV+/− precategorizations. For the postscan ratings, we analyzed the graded evaluations of trustworthiness, HIV probability, and attractiveness. An average over the two sessions was calculated for each measure. Paired sample *t* tests for comparisons between trustworthiness, HIV probability, and attractiveness ratings depending on the HIV precategorizations were calculated. For all behavioral analyses, we used IBM SPSS Statistics V27 (https://www.ibm.com/).

#### Hemodynamic activity patterns

We preprocessed and analyzed the fMRI data with SPM12 (https://www.fil.ion.ucl.ac.uk/spm/software/spm12/). Preprocessing consisted of slice-time correction to the middle slice, realignment to the mean image, with unwarping on the *y*-axis, normalization and resampling to a 3 × 3 × 3 mm voxel size, and smoothing with a 9 mm (FWHM) Gaussian kernel.

First-level analyses were based on seven GLMs. GLM1 covered the precategorization and had one regressor for high risk (HIV+) and one for low risk (HIV−) stimuli. GLM2 addressed the individual trust ratings and accordingly had one regressor for trustworthy and one for untrustworthy rated stimuli. In addition, GLM3, 4, and 5 were set up for the parametric modulation to investigate brain–behavior associations, in which one regressor contained all stimuli and was modulated by the postratings of HIV probability, attractiveness, and trustworthiness. To examine functional connectivity, GLMs 6 and 7 that were identical to GLM1 and GLM2 were set up, with the only exception being the duration of 1 s instead of 0 s. For all GLMs, there was an additional regressor representing all button presses and six motion regressors from the realignment as regressors of no interest. Furthermore, each first-level model included both sessions with session as additional regressor. The high-pass filter was always set to 256 s. We used generalized psychophysiological interactions (gPPI; McLaren et al., 2012) with the default settings provided in McLaren's gPPI toolbox, using bilateral insula as seed regions. Specifically, in the toolbox, the time series of the seed regions are extracted at a default threshold of 0.5 and multiplied with the condition-specific regressors.

At the second level, group analyses were calculated with *t* tests for the following contrasts from the first-level analyses: GLM 1 and GLM 6, HIV+, HIV−, HIV+ > HIV−, HIV− > HIV+; GLM 2 and GLM 7, untrustworthy, trustworthy, untrustworthy > trustworthy, trustworthy > untrustworthy; GLM3, 4 and 5, each stimulus event × parametric modulator (postratings of attractiveness, trustworthiness, or HIV probability). We applied Probabilistic Threshold-Free Cluster Enhancement (TFCE; Smith and Nichols, 2009) to improve sensitivity in detecting fMRI signal differences and therefore to make our second-level results more robust and reliable. Unlike traditional cluster-based methods, TFCE enhances both small, intense activations and larger, less intense clusters, providing a better balance for detecting subtle but meaningful neural responses. This is especially important for our study, in which we examine complex social-cognitive processes, where activation patterns may be distributed across various brain regions or may be present in very small brain regions. The significance level was set to *p* < 0.05 FWE corrected. In addition, exploratory analyses with a more lenient threshold of *p* < 0.001 uncorrected were conducted. ROI analyses were performed for amygdala, insula, mPFC, mOFC, Nacc, pSTS, and ACC with small-volume correction (svc) with FWE < 0.05 peak voxel correction. Cluster size was set to a standard threshold of *k* = 5 for all analyses.

The insula mask and the mPFC mask were created with MARINA (Walter, 2003). The mOFC mask was taken from the labels atlas in WFU-PickAtlas (Maldjian et al., 2003). Nacc mask was taken from automatic anatomy labeling atlas 3 (Rolls et al., 2020a,b). The amygdala mask and the ACC mask were taken from Neuromorphometrics atlas (Neuromorphometrics, as included in SPM12). The mask for the pSTS was taken from Mier et al. (2010).

## Results

### Behavior

A chi^2^ was used to compare HIV precategorization and categorical trustworthiness ratings (Table 1). Results show a significant interaction between HIV precategorization and trustworthiness in-scanner ratings (*p* < 0.001). Specifically, participants rated on average 59.44% (±14.71) of HIV+ stimuli as untrustworthy and 72.96% (±15.13) of HIV− as trustworthy.

**Table 1.** Distribution of in-scanner trustworthiness ratings in relation to the precategorization into HIV positive and HIV negative

	Trustworthiness	
	Not trustworthy	Trustworthy
HIV	+	59.8%	40.2%
	−	26.8%	73.2%

**Figure 2.**
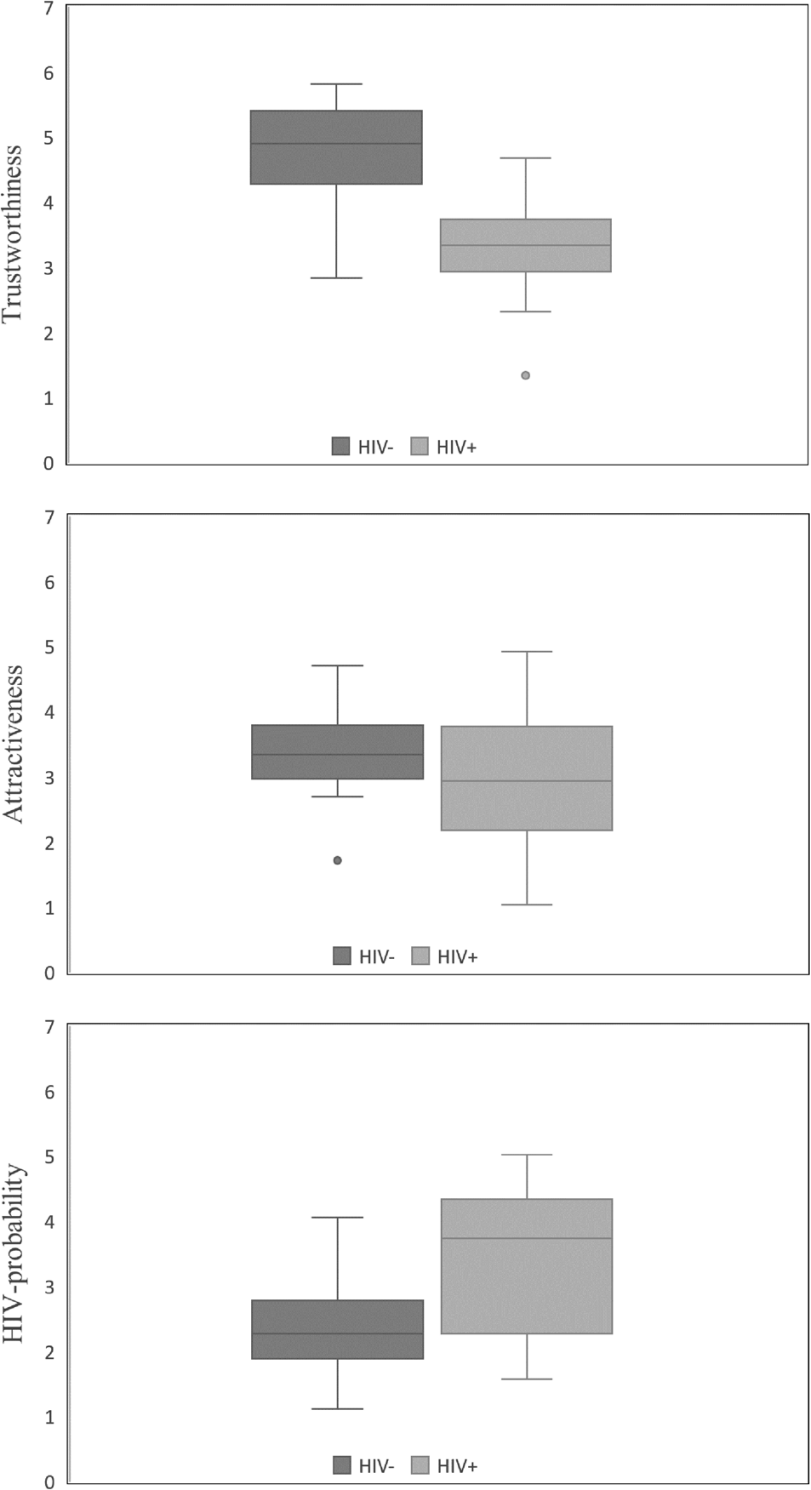
Trustworthiness, attractiveness, and HIV probability post ratings previously categorized in HIV− and HIV+.

Postratings of trustworthiness (*t*_(24)_ = 12.14, *p* < 0.001, *d* = 0.63) and attractiveness (*t*_(24)_ = 3.18, *p* = 0.004, *d* = 0.67) were significantly higher for HIV− than those for HIV+ preclassified persons (Fig. 2), and HIV probability postratings were significantly lower (*t*_(24)_ = −7.01, *p* < 0.001, *d* = 0.76; Fig. 2). Trustworthiness ratings during the fMRI measurements correlated significantly positive with trustworthiness postratings (*r* = 0.73, *p* < 0.001), on a trend level with attractiveness postratings (*r* = 0.38, *p* = 0.061), but not with HIV probability postratings (*r* = −0.33, *p* = 0.107). Trustworthiness postratings correlated significantly positive with attractiveness postratings (*r* = 0.55, *p* = 0.004), but not with HIV probability postratings (*r* = −0.33, *p* = 0.105). Attractiveness postratings did not correlate with HIV probability postratings (*r* = −0.04, *p* = 0.854).

### Confirmatory fMRI analyses

#### Analyses according to the preclassification

Comparison of HIV+ and HIV− revealed no significant results when applying FWE correction or ROI analyses, neither for activation nor for left and right insula connectivity.

#### Analyses based on individual trustworthiness ratings

**Figure 3.**
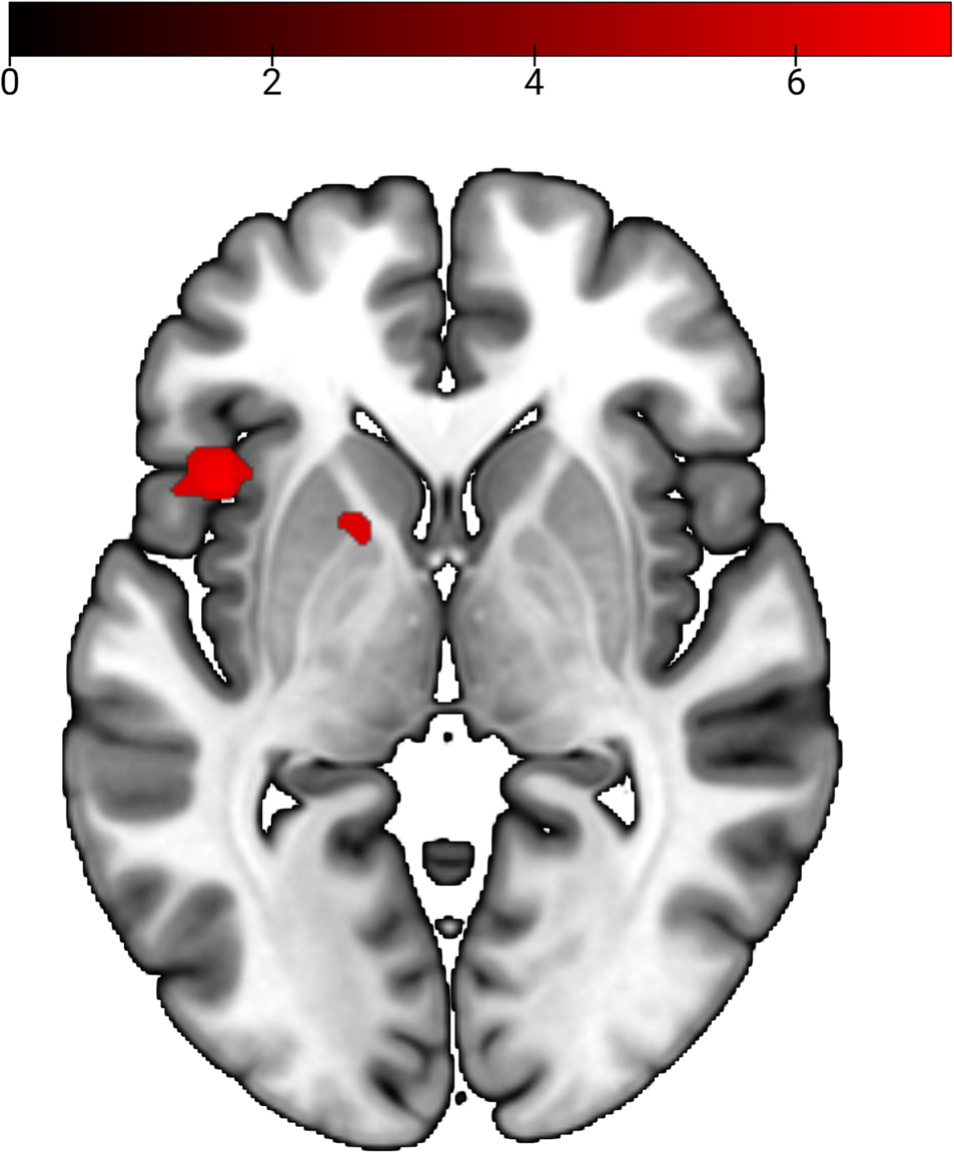
Contrast not trustworthy > trustworthy; *p* < 0.05 (FWE corrected), *k* = 5.

Whole-brain analyses of faces rated as not trustworthy (compared with trustworthy) revealed activation in left insula and left putamen, both reaching into inferior frontal cortex (Fig. 3, Table 2). For this contrast, small volume analyses showed activation in bilateral insula (left: MNI: −42, 14, −1; *T* = 7.31; *p* svc ≤ 0.001; *k* = 471; right: MNI: 42, 23, 2; *T* = 4.99; *p* svc = 0.008; *k* = 221) and left amygdala (MNI: −27, 2, −19; *T* = 4.09; *p* svc = 0.006; *k* = 12). Again, no significant left or right insula connectivity differences between conditions were found, neither in the whole brain nor in the ROI analyses.

**Table 2.** Activation in the contrast not trustworthy > trustworthy, *k* = 5

Not trustworthy > trustworthy	BA	*k*	MNI			*t* value	*p*_FWE < 0.05_
Area			*x*	*y*	*z*		
Left insular cortex	13	58	−42	14	−1	7.31	0.004
Left inferior frontal gyrus	13		−48	14	−5	7.08	0.007
Left insular cortex	13		−33	14	11	6.24	0.047
Left putamen		42	33	26	7	6.76	0.015
Left inferior prefrontal gyrus	47		39	11	29	6.72	0.016
Left inferior prefrontal gyrus	47		33	26	5	6.39	0.035

#### Brain–behavior associations

Parametric modulation of attractiveness and graded trustworthiness ratings revealed an activation cluster in right Nacc (MNI: 18, 5, −7; *T* = 3.71; *p* svc = 0.029; *k* = 59), but not in left Nacc or mOFC. No further results of the parametric modulation were significant, neither for the whole brain nor the ROI analyses; i.e., there were no significant associations between HIV probability, as well as trustworthiness ratings and brain activation.

### Exploratory fMRI analyses

#### Analyses according to the preclassification

**Figure 4.**
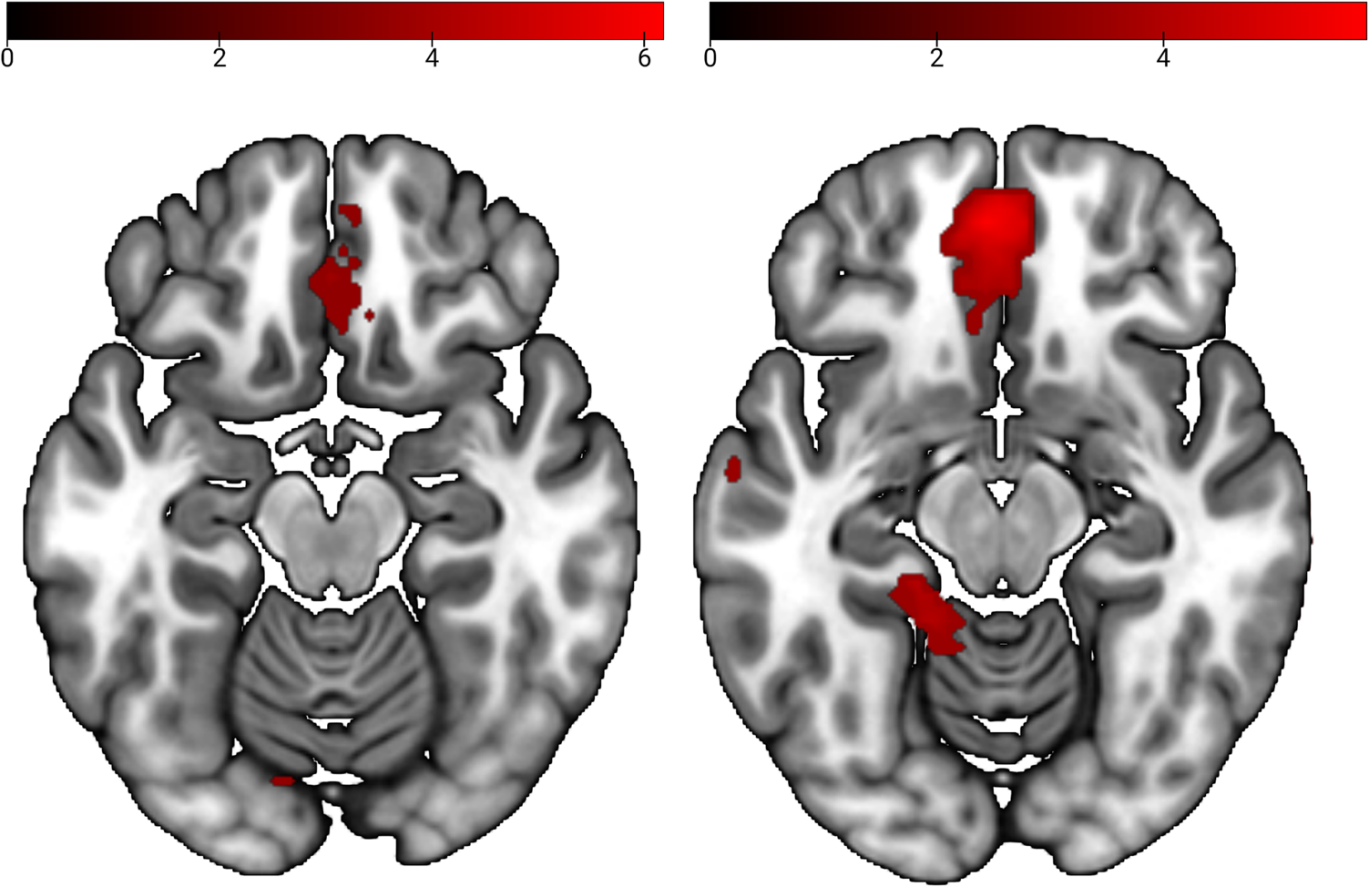
Left, Contrast trustworthy > not trustworthy; *p* < 0.001 (uncorrected), *k* = 5. Right, Contrast HIV− > HIV+; *k* = 5; *p* = 0.001 (uncorrected).

Whole-brain analyses with a liberal significance threshold (*p* < 0.001, *k* = 5) showed higher activation in mOFC, left superior frontal gyrus, and right angular gyrus for HIV− compared with HIV+ (Fig. 4, right; Table 3). Small volume analyses revealed higher activation in left pSTS (MNI: −45, −70, 20; *T* = 4.16; *p* svc = 0.037; *k* = 108), mPFC (MNI: −3, 47, −13; *T* = 5.89; *p* svc = 0.003; *k* = 328), and mOFC (left: MNI: −3, 38, −22; *T* = 5.66; *p* svc = 0.002; *k* = 18; right: MNI: 0, 44, −19; *T* = 5.63; *p* svc = 0.002; *k* = 14). Further ROI analyses as well as whole-brain analyses revealed no regions with differential activation or significantly enhanced left or right insula connectivity for HIV− versus HIV+ preclassified images and vice versa.

**Table 3.** Activation in the contrast HIV− > HIV+, *k* = 5

HIV− > HIV+	BA	*k*	MNI			*t* value	*p* < 0.001
Area			*x*	*y*	*z*		
Orbitofrontal area	11	218	−3	47	−13	5.89	<0.001
Orbitofrontal area	11		−3	38	−22	5.66	<0.001
Orbitofrontal area	11		0	29	−22	5.12	<0.001
Left inferior temporal gyrus	20	36	−60	−7	−25	5.07	<0.001
Left middle temporal gyrus	21		−60	−1	−19	4.75	<0.001
Left angular gyrus	39	64	−45	−67	23	4.39	<0.001
Left angular gyrus	39		−67	−1	−19	3.66	0.001
Right retrosplenial cingulate cortex	29	643	15	−46	5	4.38	<0.001
Left primary visual cortex	17		−12	−85	−1	4.30	<0.001
Left visual association cortex	19		−18	−43	−10	4.27	<0.001
Right angular gyrus	39	52	51	−58	23	4.22	<0.001

#### Analyses based on individual trustworthiness ratings

Additional exploratory small volume analyses for untrustworthy (compared with trustworthy) faces showed activation in bilateral Nacc (left: MNI: −18, 5, −7; *T* = 6.76; *p* svc <0.001; *k* = 81; right: MNI: 18, 11, −4; *T* = 5.52; *p* svc = 0.001; *k* = 67) and left ACC (MNI: −9. 26, 26; *T* = 4.24; *p* svc = 034; *k* = 133).

Whole-brain analyses for the reverse contrast with a more lenient significance threshold revealed activation in mOFC (Fig. 4, left; Table 4). Exploratory small volume analyses for the same contrast revealed no significant activation differences in the ROIs.

**Table 4.** Activation in the contrast trustworthy > not trustworthy, *k* = 5

Trustworthy > not trustworthy	BA	*k*	MNI			*t* value	*p* < 0.001
Area			*x*	*y*	*z*		
Left visual association cortex	18	240	−12	−82	−10	6.22	<0.001
Left lingual gyrus			−24	−73	−4	4.92	<0.001
Left lingual gyrus			−30	−73	2	3.64	0.001
Orbitofrontal area	11	13	6	53	−10	4.00	<0.001
Orbitofrontal area	11	27	0	35	−16	3.97	<0.001
Medial frontal gyrus	25		3	23	−16	3.87	<0.001

## Discussion

In the present fMRI study, participants judged the trustworthiness of individuals preclassified as having high or low HIV probability. We investigated how the participants’ judgments of trustworthiness, HIV risk, and attractiveness are interrelated and associated to activation of regions involved in salience and trust perception, specifically dACC, insula, mPFC, Nacc, as well as amygdala and pSTS. In addition, we analyzed functional connectivity of the bilateral insulae.

Persons preclassified as HIV−, i.e., less likely to be infected with HIV, were rated more often as trustworthy than persons previously classified as HIV+. This could be interpreted as hinting toward a cross-sample perception of other persons’ safety. Also, in the postratings with graded evaluations, trustworthiness ratings were higher and HIV probability was lower, for the HIV− than HIV+ stimuli. fMRI-based cortical activity both upon preclassification and upon trustworthiness evaluation (albeit only with an uncorrected significance threshold) indicated activation in mOFC. This mOFC response seems not to derive from a joint representation of attractiveness and trustworthiness, because attractiveness ratings were associated with Nacc, but not with mOFC activation. Rather, we conclude that for persons perceived as trustworthy and safe (i.e., individually evaluated as trustworthy and precategorized as HIV−), the mOFC response may signal a rewarding property (Rolls et al., 2020a). For the persons preclassified as HIV+, the agreement between in-scanner trustworthiness and prior HIV ratings was ∼60%, suggesting a higher agreement of trustworthiness and HIV− classifications (>70% agreement) than of untrustworthiness and HIV+ classification. While this does not necessarily reflect the objective trustworthiness of a person, it indicates that the perception of safety going along with mOFC response has higher convergence across study samples than the perception of riskiness.

Importantly, while attractiveness ratings did not differ between HIV+ and HIV−, attractiveness and trustworthiness were associated. Previous studies have shown a (complex) relationship between ratings of attractiveness and trustworthiness (Gutierrez-Garcia et al., 2019; Li and Liu, 2021), but not between attractiveness and HIV probability (Schmälzle et al., 2011). In contrast to earlier findings that showed a relationship between attractiveness perception and probability of careless (unprotected) sexual behavior (Eleftheriou et al., 2016, 2019), our results suggest that the attraction to another person is related to their perceived trustworthiness but seems not to lead to a faulty perception of safety with regard to STI.

Although the behavioral ratings and the activation in mOFC suggest an association between preclassified HIV probability and individual trustworthiness ratings, only individual ratings (as untrustworthy), but not preclassification (as HIV+), resulted in activation of the SN and related regions involved in salience and trust processing. Facial stimuli rated as untrustworthy in comparison with faces rated as trustworthy were associated with activity in bilateral insula, bilateral Nacc, left ACC, and left amygdala. In agreement with meta-analytical findings (Bzdok et al., 2011), this demonstrates a prominent role of the amygdala for trustworthiness judgments. The meta-analysis of Bzdok and colleagues (Bzdok et al., 2011) also shows activation in pSTS for trustworthiness tasks. The pSTS is generally involved in social perception and interaction (Hein and Knight, 2008; Deen et al., 2015) but seems not to be differentially activated by trustworthy versus untrustworthy faces. As pSTS activation, however, was enhanced in response to faces preclassified as HIV− in contrast to HIV+, the pSTS might play a particular role in safety perception. However, this finding warrants replication, before firm conclusions can be drawn. While Häcker and colleagues reported enhanced insula and mPFC activation for persons rated as HIV+, we found higher insula, ACC, Nacc, and amygdala activation for untrustworthy versus trustworthy ratings. Insula, amygdala, ACC, and Nacc are involved in (social) salience processing (Menon and Uddin, 2010; Schmidt et al., 2019). As Häcker and colleagues propose (Häcker et al., 2015), participants have intuitive sensing of risk; i.e., a “HIV risk feeling.” As activity in insula, but not connectivity between insula and mPFC, differed between persons rated as trustworthy versus untrustworthy, the regional activity of certain structures within the SN seems modulated by such a risk feeling, but not the communication between the selected structures. The lack of connectivity results from our data stands in contrast to previous studies that reported enhanced functional connectivity dependent on HIV categorization (Renner et al., 2012; Häcker et al., 2015; Schmälzle et al., 2019b).

Across various analyses of the here presented data (both for HIV+ vs HIV− faces and trustworthy vs untrustworthy faces), there is a notable absence of significant insula connectivity differences between conditions. This could imply that the difference in processing risk-related social information within our task is more localized to specific brain regions rather than involving broader neural networks. However, a more likely explanation for our lack of connectivity findings might be our selection of the seed region rather than an absence of differential connectivity effects. Specifically, our choice of the insula as seed region was based on studies focusing on perceived HIV risk in the same stimulus set (Häcker et al., 2015). The insula is involved in interoception, emotional awareness, risk perception, and certain aspects of emotion processing, such as disgust and fear. In addition, it is often activated when processing emotionally salient or risky stimuli (Uddin et al., 2017), which makes it a reasonable candidate for examining responses to faces associated with HIV risk and low trust. While we find insula activation when persons were perceived as not trustworthy, connectivity might not change between not trustworthy and trustworthy/HIV+ and HIV− perception. Possibly, trustworthiness judgments may be related more strongly to variation in networks involved in social reward evaluation and value-based decisions. Relevant regions of interest that may serve as seed regions for future connectivity analyses in the context of trustworthiness are mPFC, amygdala, OFC, and even the Nacc.

To our knowledge, our study is the first attempt to differentiate SN activity for faces preclassified as HIV+ versus HIV−. In our study, insula, ACC, Nacc, and amygdala were only related to untrustworthiness ratings, but not to HIV+ preclassification. Since we applied an implicit risk assessment by participants rating trustworthiness and not HIV probability during fMRI, we cannot draw conclusions about an involvement of the SN in individual HIV judgments as Häcker et al. (2015). The discrepancy in SN activation in our study between individual trustworthiness ratings and precategorized HIV probability emphasizes the importance of individual trustworthiness perception. In contrast to the behavioral findings, the fMRI findings suggest a reduced transferability of HIV stereotypes to individual salience signals of trustworthiness.

Such SN activation patterns upon facial images judged as untrustworthy may have evolutionary roots. Rapid judgment of social counterpart's reduced trustworthiness and their risk of being a life-endangering disease carrier should ignite the brain's alertness response and prepare quick (risk–caution) decisions, thus bearing survival advantage. Even though average ratings indicate prototypic features that are related to trustworthiness perception (Todorov et al., 2008; Sessa and Meconi, 2015), our findings suggest that the features of presented facial images activate the alarm system seems to be highly individual in nature. Importantly, these intuitive perceptions might result in false risk and/or safety signals. We had neither objective information about the general trustworthiness of our stimulus persons nor their health status.

### Limitations

Several limitations of our study have to be noted. First, the stimulus persons were shown in different naturalistic contexts. Although this makes the pictures less artificial, different contexts may have influenced the trustworthiness ratings. In addition, there was no matching for arousal and valence for HIV+ and HIV− stimuli. Matching the stimulus material with regard to these characteristics would have been detrimental to the manipulation of the independent variable and would have reduced the probability of finding differences in trustworthiness ratings between HIV+ and HIV− precategorized persons. By definition, persons perceived as not trustworthy elicit arousal and negative valence, while persons perceived as trustworthy should be rather related to positive valence and limited arousal. Second, participants were not provided with a definition or contextualization of trustworthiness prior to the experiment, which may have increased the interindividual variability of subjective definitions. To address this, we specified the context for the trustworthiness judgments in a follow-up study (Wolber et al., in preparation) and mentioned that participants should imagine a dating context.

Third, six participants had to be excluded due to a positive bias; i.e., not rating any of the stimulus persons as untrustworthy in at least one of the sessions which is an interesting finding itself. This, however, resulted in a final sample of 25 participants that is rather small for current standards in fMRI studies and which limits the reliability of the results. To counteract, we combined the sessions that were 4–5 weeks apart in our analyses, since it was shown that aggregating data from multiple sessions can increase reliability (Noble et al., 2017).

A fourth and most important limitation is the partial stimulus overlap in the “preclassified” and “individual rating” analyses (59.8% for HIV+ and untrustworthy judgments, 73.2% for HIV− and trustworthy judgments). Both analyses share individually differing proportions of stimulus materials. Thus, a similarity in neural activation for preclassification and individual trustworthiness analyses that occurred for the persons rated as trustworthy seems logical. Since we used the same stimulus set for both sessions, independent analyses are not possible. Future studies may investigate both concepts with two stimulus sets with two sessions and analyze individual ratings in session one with stimulus set one and precategorization in session two stimulus set two or might ask in session one for trustworthiness and in session two for HIV probability.

### Conclusion

Taken together we found within-sample congruence for trustworthiness and HIV perception, while across-sample congruence was higher for trustworthiness and HIV− than for untrustworthiness and HIV+ judgments. This was reflected in mOFC activation during HIV− preclassified images and individual trustworthiness ratings, and regions involved in salience and trust processing for individual untrustworthy-ratings, but not HIV+ preclassification. These findings suggest cross-sample agreement of safety perception in behavior and brain activation but highlight the importance of individuality of risk perception.

## Synthesis

Reviewing Editor: Catherine Schevon, Columbia University Decisions are customarily a result of the Reviewing Editor and the peer reviewers coming together and discussing their recommendations until a consensus is reached. When revisions are invited, a fact-based synthesis statement explaining their decision and outlining what is needed to prepare a revision will be listed below. The following reviewer(s) agreed to reveal their identity: Paul Regier.

We base our decision on the comments of one external reviewer and our own evaluation since we were not able to secure a second reviewer and do not want to further delay the decision. The one reviewer has a number of important comments that need to be carefully addresses in a revised version.

Reviewer's comments:

The study investigates the neural correlates of trustworthiness in people pre-categorized as either having high or low HIV risk. Stimuli previously categorized as HIV+ were perceived as less trustworthy and with more HIV probability. The primary neural results show anterior insula activation is associated with perception of untrustworthy (vs. trustworthy) faces. Exploratory results suggest higher activation in mOFC to those pre-classified as HIV+, and that there was more activation in the salience network and associated regions to untrustworthy faces. The study has several technical and statistical issues that limit enthusiasm for publication at this time.

Main issues:

1a) For the primary analysis, the authors state that they set the voxel-level threshold to p < 0.05 and the cluster-level threshold to k = 5. It is unclear how the k=5 level was determined (e.g., there is no mention of software or techniques to calculate the cluster-level threshold). If the k=5 level was derived from an established algorithm, then the primary results should only include the untrustworthy > trustworthy contrast, with results in the anterior insula.

1b) No other significant results should be reported for primary findings, as the rest would be reported as exploratory in a different section. This would include ROI results, since it seems the small-volume corrections were uncorrected, as well.

1c) To this reviewer, the whole-brain analysis does not make sense, given the focus on several a priori regions in the introduction. Given the high-degree of multiple comparisons at the whole-brain level and the small sample size, a more focused hypothesis-based approach seems justified. And while the introduction does provide a path to this hypothesis-based approach, the statistics are a bit disordered. Statistical approaches include pFWE p < 0.05, uncorrected p < 0.001, uncorrected p = 0.001, and uncorrected p < 0.05 (SVC), all with k = 5. These results are all reported in the same paragraph and in the same figure, which lends a similar similar weight to each result, when most of the results reported would be considered exploratory or hypothesis-generating.

2) Another issue is the use of faces that were previously-categorized as high or low HIV risk (per Hacker et al., 2015). The use of these images is stated as a novel factor in the abstract and introduction; however, it is unclear how or why someone would be labeled as such, except that these images were included in a study where participants were required to categorize pictures of people as high or low HIV. Using these images with a new set of participants seems problematic. There is no rationale (stated or otherwise) that would suggest that anyone previously perceived to have HIV would be labeled similarly by a new group of participants. The fact that participants did tend to rate (60%) those previously labeled as high HIV risk as higher HIV-probability in the current study is interesting but could be completely random.

3a) Together, the results suggest brain activation in one region of the salience network (insula) to individuals perceived as untrustworthy but that this seems to have little to do with HIV status. This would detract from the novelty factor, but is still an interesting result.

3b) The lack of a (primary) HIV-related result could be due to use of images with pre-categorized labels. It would be interesting to use the HIV-probability scores to create new HIV+ and HIV- contrasts and re-analyze the data with categorizations that were created by current participants. In the limitations, this is even alluded to in that the authors say "Future studies may investigate both concepts with independent stimulus sets, for example by running two sessions and analyzing individual ratings in one session and pre-categorization in the other session." The authors do have access to 2 sessions, so it seems like this could be done.

4) It is unclear why data from both sessions are being used in this study, and there is no data on the changes from session 1 to 2 on any of the measures. In addition, it is unclear whether fMRI data has been averaged over these sessions like they were for the behavioral data. In the limitations, it is stated that sessions were combined, but it is unclear how. Further, it is unclear why paired t-tests and McNemar tests are being used on behavioral data that has been averaged over the sessions - which should mean there is only 1 data-point per person per measure - so no repeated measures should be occurring.

Minor issues

5) The functional connectivity analysis is underdeveloped and seems unnecessary.

6) The task is called an STI task, but it's just a task which shows faces and asks participants to label them as trustworthy or not.

7) In the limitations, the authors say, "the instruction for future studies might be specified to telling the participants that the stimuli were selected such that 50% of them fall in one category and 50% in the other". However, the chosen stimuli were already arbitrarily labeled as such previously, and this would just bias their responses more.

8) Line 56 is missing a reference.

9) Authors mention BART a few times, but nothing is reported on this task.

10) Lines 121 and 122 reference unfinished work.

11) Did participants need to be excluded from all contrasts or just the trustworthy ones? There would be no trustworthy > untrustworthy contasts for those individuals; however, there would still be HIV+ > HIV- contrasts.

12) Probabilistic Threshold free Cluster Enhancement could use a better explanation/rationale.

13) Line 205 suggests participants correctly labeled stimuli as untrustworthy, but this is an arbitrary rating with no inherent value.

14) Results could be driven by attractiveness, since this is correlated with trustworthiness.

15) It would be helpful to see the actual stimuli. Perhaps a database that is accessible to reviewers for the review process would be warranted.

## Author Response

Wednesday, 04 December 2024 Dear Prof. Michel, We would like to thank you and the reviewer for the careful and detailed review of our Manuscript "Are you safe or should I go? How perceived trustworthiness and probability of a sexual transmittable infection impact activation of the salience network", which we have revised according to your and the reviewer's suggestions and now resubmit for your consideration for publication in eNeuro.

We are grateful to you and the reviewer for the valuable comments. We believe that we have been able to address all issues and that the manuscript has improved as a result. We summarize our responses to the reviewer below and all changes made in the manuscript on an item-by-item basis. For easier reference, copied text from the manuscript is presented in italics and revisions are marked yellow.

We appreciate the opportunity to incorporate these suggestions into our manuscript. Please do not hesitate to contact us if we can provide further clarification. We will be happy to consider any further changes that you or the reviewer may deem necessary.

On behalf of the authors, Yours sincerely, Alexander Wolber - Response Letter We are indebted to the editor and the reviewer for their very helpful suggestions. We believe that incorporating their recommendations strengthened the manuscript significantly. In the following we reply to their recommendations and questions point-by-point. Reviewer comments are presented in bold. Copied text from the manuscript is presented in italics and revisions are marked yellow.

1a) For the primary analysis, the authors state that they set the voxel-level threshold to p < 0.05 and the cluster-level threshold to k = 5. It is unclear how the k=5 level was determined (e.g., there is no mention of software or techniques to calculate the cluster-level threshold). If the k=5 level was derived from an established algorithm, then the primary results should only include the untrustworthy > trustworthy contrast, with results in the anterior insula.

1b) No other significant results should be reported for primary findings, as the rest would be reported as exploratory in a different section. This would include ROI results, since it seems the small-volume corrections were uncorrected, as well.

1c) To this reviewer, the whole-brain analysis does not make sense, given the focus on several a priori regions in the introduction. Given the high-degree of multiple comparisons at the whole-brain level and the small sample size, a more focused hypothesis-based approach seems justified. And while the introduction does provide a path to this hypothesis-based approach, the statistics are a bit disordered. Statistical approaches include pFWE p < 0.05, uncorrected p < 0.001, uncorrected p = 0.001, and uncorrected p < 0.05 (SVC), all with k = 5. These results are all reported in the same paragraph and in the same figure, which lends a similar weight to each result, when most of the results reported would be considered exploratory or hypothesis-generating.

We reply to comments 1a-c together, because they all relate to our results presentation.

We thank the reviewer for pointing out the lack of clarity. We did not use software for determining our cluster-level threshold, but used a commonly applied threshold that on the one hand avoids reporting noisy activation of single voxels, and on the other hand allows to reveal activation in small regions (e.g. Nucleus accumbens). We mention now in the revised manuscript that the cluster size threshold was set to a standard threshold. We also thank the reviewer for the suggestion to reorganize our results. We completely revised the results section and separated the section in which we report our fMRI findings into a "confirmatory fmri analyses" and an "exploratory fMRI analyses" paragraph for better differentiation of hypothesis-driven and exploratory analyses. We also adjusted the figures and tables and the supplementary section accordingly. In the confirmatory section, we report the whole-brain results with a family-wise error correction, and the region of interest analyses with a directed hypothesis. In the exploratory section, we report the whole results without family-wise error correction, and further region of interest results without a directed hypothesis.

We want to mention that we applied small volume correction for our region of interests; i.e. uncorrected significance threshold, but family-wise error correction for the peak voxel. We changed the wording in the revised manuscript to avoid ambiguity of the applied significance threshold for the small volume correction.

Page 6 and 7 for reorganization in confirmatory and exploratory section "3.2 Confirmatory fMRI Analyses 3.2.1.1. Analyses according to the pre-classification Comparison of HIV+ and HIV- revealed no significant results when applying FWE-correction or ROI analyses, neither for activation nor for left and right insula connectivity.

3.2.1.2. Analyses based on individual trustworthiness ratings Whole brain analyses of faces rated as not trustworthy (compared to trustworthy) revealed activation in left insula and left putamen, both reaching into inferior frontal cortex (see Fig. 3 and Table 3)). For this contrast, small volume analyses showed activation in bilateral insula (left: MNI: -42, 14, -1; T = 7.31; p svc {less than or equal to} .001; k = 471; right: MNI: 42, 23, 2; T = 4.99; p scv = .008; k = 221) and left amygdala (MNI: -27, 2, -19; T = 4.09; p svc = .006; k = 12). Again, no significant left or right insula connectivity differences between conditions were found, neither in the whole brain, nor in the ROI analyses.

3.2.2. Brain behavior associations Parametric modulation of attractiveness and graded trustworthiness ratings revealed an activation cluster in right Nacc (MNI: 18, 5, -7; T = 3.71; p svc = .029; k = 59), but not in left Nacc or mOFC. No further results of the parametric modulation were significant, neither for the whole brain, nor the ROI analyses; i.e. there were no significant associations between HIV probability, as well as trustworthiness ratings and brain activation.

3.3. Exploratory fMRI analyses 3.3.1.1. Analyses according to the pre-classification Whole brain analyses with a liberal significance threshold (p < 0.001, k = 5) showed higher activation in mOFC, left superior frontal gyrus and right angular gyrus for HIV- compared to HIV+ (see Fig. 4 right and table 2). Small volume analyses revealed higher activation in left pSTS (MNI: -45, -70, 20; T = 4.16; p svc = 0.037; k = 108), mPFC (MNI: -3, 47, -13; T = 5.89; p svc = 0.003; k = 328), and mOFC (left: MNI: -3, 38, -22; T = 5.66; p svc = 0.002; k = 18; right: MNI: 0, 44, -19; T = 5.63; p svc = 0.002; k = 14). Further ROI analyses, as well as whole brain analyses revealed no regions with differential activation, or significantly enhanced left or right insula connectivity for HIV- versus HIV+ pre-classified images and vice versa.

3.3.1.2. Analyses based on individual trustworthiness ratings Additional exploratory small volume analyses for untrustworthy (compared to trustworthy) faces showed activation in bilateral Nacc (left: MNI: -18, 5, -7; T = 6.76; p svc <.001; k = 81; right: MNI: 18, 11, -4; T = 5.52; p svc = .001; k = 67) and left ACC (MNI: -9. 26, 26; T = 4.24; p svc = 034; k = 133).

Whole brain analyses for the reverse contrast with a more lenient significance threshold revealed activation in mOFC (see Fig. 4 left and Table 4). Exploratory small volume analyses for the same contrast revealed no significant activation differences in the ROIs." Line 203 - 206 for FWE-correction of all small volume analyses "ROI analyses were performed for amygdala, insula, mPFC, mOFC, Nacc, pSTS and ACC with small-volume correction (svc) with FWE < .05 peak voxel correction. Cluster size was set to a standard threshold of k = 5 for all analyses." 2) Another issue is the use of faces that were previously-categorized as high or low HIV risk (per Hacker et al., 2015). The use of these images is stated as a novel factor in the abstract and introduction; however, it is unclear how or why someone would be labeled as such, except that these images were included in a study where participants were required to categorize pictures of people as high or low HIV. Using these images with a new set of participants seems problematic. There is no rationale (stated or otherwise) that would suggest that anyone previously perceived to have HIV would be labeled similarly by a new group of participants. The fact that participants did tend to rate (60%) those previously labeled as high HIV risk as higher HIV-probability in the current study is interesting but could be completely random.

We thank the reviewer for pointing out that we need to explain why we assumed to find that the HIV categorizations could be reliably transferred to our sample. The stimulus materials were used in three previous studies of the research groups of Renner und Schupp (Schmälzle et a., 2011, Renner et al., 2012, Häcker et al., 2015). All three studies state that there are no visual characteristics of HIV infection and that the participants probably make these decisions based on characteristics, which are described by the authors as "high-risk stereotypes". Importantly, in the paper by Renner et al., 2012, two subsamples were collected and the reliability of the ratings of the stimulus material was examined. The authors demonstrated high retest-reliability and high cross-sample agreement. In our study, we are relying on these previous results. We apologize for not having mentioned these results on the cross-sample agreement explicitly in the previous version of the manuscript. We believe it underlines our approach and our reasoning that we can replicate earlier findings with the stimulus materials.

Line 80 - 82 "Renner et al. (2012) demonstrated high inter-rater agreement and high test-retest reliability of HIV probability ratings of the stimulus materials, suggesting the replicability." 3a) Together, the results suggest brain activation in one region of the salience network (insula) to individuals perceived as untrustworthy but that this seems to have little to do with HIV status. This would detract from the novelty factor, but is still an interesting result.

We fully agree. While subjective trust perception correlates negatively with HIV-ratings as shown by Renner et al., 2012, and also in our study, this finding is not reflected in brain activation. This is true for the analyses according to pre-classification as well as for the parametric modulations.

We added to the revised discussion that we cannot draw conclusions about SN activity and individual HIV probability judgements in our study.

Line 341 - 347 "To our knowledge, our study is the first attempt to differentiate SN activity for faces pre-classified as HIV+ versus HIV-. In our study, Insula, ACC, Nacc and amygdala were only related to untrustworthiness ratings, but not to HIV+ pre-classification. Since we applied an implicit risk assessment by participants rating trustworthiness and not HIV probability during fMRI, we cannot draw conclusions about an involvement of the SN in individual HIV judgements as Häcker and colleagues (2015). The discrepancy in SN activation in our study between individual trustworthiness ratings and pre-categorized HIV probability emphasizes the importance of individual trustworthiness perception. In contrast to the behavioral findings, the fMRI findings suggest a reduced transferability of HIV stereotypes to individual salience signals of trustworthiness." 3b) The lack of a (primary) HIV-related result could be due to use of images with pre-categorized labels. It would be interesting to use the HIV-probability scores to create new HIV+ and HIV- contrasts and re-analyze the data with categorizations that were created by current participants. In the limitations, this is even alluded to in that the authors say "Future studies may investigate both concepts with independent stimulus sets, for example by running two sessions and analyzing individual ratings in one session and pre-categorization in the other session." The authors do have access to 2 sessions, so it seems like this could be done.

We would like to thank you for the idea using the present ratings to create new contrasts and to use the two sessions for different analyses. However, to our regret we deem it as not feasible with our data. This has two main reasons. 1. We already analyzed the association of individual HIV probability post-ratings and brain activation with parametric modulation which is presented in the original manuscript. We found no significant association, neither with family-wise error correction, nor with the exploratory threshold of p < 0.001. Thus, an additional analysis with binary categorization of the dimensional post-ratings would lose the variance that is reflected in the parametric modulation and would likely reveal even weaker results. 2. The pre-classification and the subjective ratings are based on the same stimulus set and the same stimuli were used for both sessions. An analysis of the subjective ratings in session one and pre-classification in session two (or dichotomized HIV probability post-ratings) should not reveal substantially other results than with the parametric modulation or pre-classification within the combined data set. The problem that the same stimulus persons with the same trustworthiness-rating were applied in both sessions cannot be circumvented by analyzing the sessions separately. Given the regressor overlap between pre-classification and trustworthiness-ratings, separating the sessions unfortunately does not avoid this problem.

In another new study, two stimulus sets could be used and the question on HIV could be asked in one session and the question on trustworthiness in another session. This would additionally avoid that participants are explicitly thinking about the same concept (i.e. trustworthiness in our current study) in both sessions. Then, via parametric modulation of the post-rating, including the neural activation of both sessions, one might gain even further insight into the specific differences or similarities of trustworthiness and HIV focus.

We revised the description of the research idea with two independent stimulus sets to make our reasoning clearer and also to explain why this is unfortunately not possible in the current study.

Line 383 - 388 "Thus, a similarity in neural activation for pre-classification and individual trustworthiness analyses that occurred for the persons rated as trustworthy seems logical. Since we used the same stimulus set for both sessions, independent analyses are not possible. Future studies may investigate both concepts with two stimulus sets with two sessions and analyze individual ratings in session one with stimulus set one and pre-categorization in session two stimulus set two, or might ask in session one for trustworthiness and in session two for HIV probability." 4) It is unclear why data from both sessions are being used in this study, and there is no data on the changes from session 1 to 2 on any of the measures. In addition, it is unclear whether fMRI data has been averaged over these sessions like they were for the behavioral data. In the limitations, it is stated that sessions were combined, but it is unclear how. Further, it is unclear why paired t-tests and McNemar tests are being used on behavioral data that has been averaged over the sessions - which should mean there is only 1 data-point per person per measure - so no repeated measures should be occurring.

Thank you for pointing out the unclear wording in the manuscript. fMRI data from both sessions were combined in one first-level model with session as covariate. The behavioral data was averaged across the two sessions. Originally, we conducted two sessions to allow assessment of retest-reliability. A manuscript regarding task-reliability is currently in preparation. We decided to analyze both sessions together in the current paper to increase the number of trials per condition and with this to enhance reliability.

Line 185 and 186 "Furthermore, each first level model included both sessions with session as additional regressor." We are indebted to the reviewer for asking about the McNemar test. This was a mistake, because we used a standard Chi2-test. Since the in-scanner ratings are relative frequencies, we summed both sessions up and calculated a Chi² test to give both sessions equal weight. This careless error was corrected in the revised manuscript.

The post-ratings were averaged over both sessions. Afterwards mean differences between the (averaged) post-ratings of HIV-probability, attractiveness and trustworthiness depending on the HIV pre-categorization were then calculated. This is the reason for the paired t-tests.

We changed the methods section accordingly.

Line 162 - 169 "For the FaRiP-task, we determined the number of in-scanner facial stimuli categorized as trustworthy (yes/no) and calculated the sum of both sessions. A Chi² test was performed for yes/no categorization relative to the HIV +/- pre-categorizations. For the post-scan ratings, we analyzed the graded evaluations of trustworthiness, HIV probability, and attractiveness. An average over the two sessions was calculated for each measure. Paired sample t-tests for comparisons between trustworthiness, HIV-probability and attractiveness ratings depending on the HIV pre-categorizations were calculated. For all behavioral analyses, we used IBM SPSS Statistics V27 (https://www.ibm.com/)." 5) The functional connectivity analysis is underdeveloped and seems unnecessary.

We thank the reviewer for directing our attention to this. We included the connectivity analysis because it was expected based on previous publications, and thus part of our preregistration. However, our dataset revealed no interesting connectivity patterns. Of course, it is essential to interpret this null-finding appropriately in the discussion.

And accordingly added a full paragraph to the discussion:

Line 317 - 337 "The lack of connectivity results from our data stands in contrast to previous studies that reported enhanced functional connectivity dependent on HIV categorization (Renner et al., 2012; Schmälzle, Hartung, et al., 2019).

Across various analyses of the here presented data (both for HIV+ vs. HIV- faces and trustworthy vs. untrustworthy faces), there is a notable absence of significant insula connectivity differences between conditions. This could imply that the difference in processing risk-related social information within our task is more localized to specific brain regions rather than involving broader neural networks. However, a more likely explanation for our lack of connectivity findings might be our selection of the seed region rather than an absence of differential connectivity effects. Specifically, our choice of the insula as the seed region was based on studies focusing on perceived HIV risk in the same stimulus set. The insula is involved in interoception, emotional awareness, risk perception, and certain aspects of emotion processing, such as disgust and fear. In addition, it is often activated when processing emotionally salient or risky stimuli (Uddin et al., 2017), which makes it a reasonable candidate for examining responses to faces associated with HIV risk and low trust. While we find insula activation when persons were perceived as not trustworthy, connectivity might not change between not trustworthy and trustworthy / HIV+ and HIV- perception. Possibly, trustworthiness judgements may be related more strongly to variation in networks, involved in social reward evaluation and value-based decisions. Relevant regions of interest, that may serve as seed regions for future connectivity analyses in the context of trustworthiness are medial prefrontal cortex (mPFC), amygdala, orbitofrontal cortex (OFC), and even the Nacc." In addition, we made two more small adjustments:

Line 269 "In addition, we analyzed functional connectivity of the bilateral insulae." Line 85 - 87 "For the fMRI measurements, we expected that HIV+ compared to HIV- stimuli are related to higher activation in amygdala, insula and mPFC, and higher connectivity between insula and mPFC." 6) The task is called an STI task, but it's just a task which shows faces and asks participants to label them as trustworthy or not.

We fully agree. The task measures a person's implicit perception of a health risk to themselves by (the faces of) other persons. We labelled the task STI task, because of the stimulus selection. We prefer to not call it trust task, because the purpose of the tasks goes beyond trust perception to the perception of (health) risks. However, in response to the reviewer's doubts, we call it now facial risk perception task (FaRIP). We are grateful for the reviewer pointing this out, because we believe that the new name presents a better description of the task.

7) In the limitations, the authors say, "the instruction for future studies might be specified to telling the participants that the stimuli were selected such that 50% of them fall in one category and 50% in the other". However, the chosen stimuli were already arbitrarily labeled as such previously, and this would just bias their responses more.

We explained in the response above how the stimuli were selected, which was based on previous studies that showed cross-group agreement. However, we fully agree that this instruction might bias the participants and removed this part from the revised manuscript.

8) Line 56 is missing a reference.

We added the reference. Thank you for the advice.

Line 55 - 57 "The salience network (SN) is a large scale brain network responding to emotional and social cues, including threatening events, such as potential health risks (Ince et al., 2023)" 9) Authors mention BART a few times, but nothing is reported on this task.

The BART is reported in detail in the publication by Schmidt et al., 2024. We mention the task to comprehensively present what the participants participated in during the measurement. We added a reference, please also see next response.

10) Lines 121 and 122 reference unfinished work.

We thank the reviewer for mentioning it. The article is meanwhile published in Psychophysiology, we accordingly revised the sentence.

Line 123 and 124 "The BART results are presented in Schmidt et al., (2024), and the findings on tasks reliability will be presented elsewhere." 11) Did participants need to be excluded from all contrasts or just the trustworthy ones? There would be no trustworthy > untrustworthy contrasts for those individuals; however, there would still be HIV+ > HIV- contrasts.

To avoid differences in results between the contrasts due to differences in included participants, we excluded the participants who were excluded for untrustworthy > trustworthy also for HIV+> HIV-. This is now explicitly mentioned in the revised manuscript.

Line 111 and 112 "Thus, the final sample that was included for all analyses consists of 25 participants (7 man, age M = 23.16; SD = 2.84 years)." 12) Probabilistic Threshold free Cluster Enhancement could use a better explanation/rationale.

We thank the reviewer for the suggestion. We extended the sentence that mentions our use of pTFCE.

Line 195 - 201 "We applied Probabilistic Threshold-Free Cluster Enhancement (TFCE) (Smith &Nichols, 2009) to improve sensitivity in detecting fMRI signal differences, and therefore to make our second-level results more robust and reliable. Unlike traditional cluster-based methods, TFCE enhances both small, intense activations and larger, less intense clusters, providing a better balance for detecting subtle but meaningful neural responses. This is especially important for our study, in which we examine complex social-cognitive processes, where activation patterns may be distributed across various brain regions or may be present in very small brain regions." 13) Line 205 suggests participants correctly labeled stimuli as untrustworthy, but this is an arbitrary rating with no inherent value.

We thank the reviewer for pointing out this issue, and agree that there is no objective right or wrong (and if there would be one, it would be more likely that the participants were wrong when judging someone as HIV positive). We present the analysis to show the extent to which the participants' trust rating was in alignment with the pre-categorization. We deleted "(correctly)" from the revised manuscript.

14) Results could be driven by attractiveness, since this is correlated with trustworthiness.

We fully agree with your line of thinking. Attractiveness plays an important role in the evaluation of trustworthiness. In the parametric modulation, we were able to show that attractiveness is related to the judgment that a person is trustworthy via the nucleus accumbens. However, there was no association between attractiveness ratings and activation in the insula. Thus, it can be speculated that attractiveness relates to a rewarding signal in the brain, but seems not to be related to the insula that was linked to the perception of others as not trustworthy.

15) It would be helpful to see the actual stimuli. Perhaps a database that is accessible to reviewers for the review process would be warranted.

Since we are not allowed to share the stimuli publicly, we added examples for both gender. These examples are for the internal review process, and not for publication.

Stimuli previously categorized HIV- :

Stimuli previously categorized HIV+ :
